# Evaluation of plant biomass resources available for replacement of fossil oil

**DOI:** 10.1111/j.1467-7652.2009.00482.x

**Published:** 2010-04

**Authors:** Robert J Henry

**Affiliations:** Bioenergy Research Institute, Centre for Plant Conservation Genetics, Southern Cross UniversityLismore, NSW, Australia

**Keywords:** bioenergy, biomass, biomaterials, plant diversity, domestication, biotechnology

## Abstract

The potential of plants to replace fossil oil was evaluated by considering the scale of production required, the area of land needed and the types of plants available. High yielding crops (50 tonnes/ha) that have a high conversion efficiency (75%) would require a global land footprint of around 100 million ha to replace current (2008) oil consumption. Lower yielding or less convertible plants would require a larger land footprint. Domestication of new species as dedicated energy crops may be necessary. A systematic analysis of higher plants and their current and potential uses is presented. Plant biotechnology provides tools to improve the prospects of replacing oil with plant-derived biomass by increasing the amount of biomass produced per unit area of land and improving the composition of the biomass to increase the efficiency of conversion to biofuel and biomaterials. Options for the production of high value coproducts and the expression of processing aids such as enzymes in the plant may add further value to plants as bioenergy resources.

## Introduction

Biomaterials and bioenergy have long been produced from plants. The development of oil from fossil fuel replaced many of these traditional uses during the twentieth century. The prospect of oil supplies being exhausted and concern about the impact on the atmosphere of adding the carbon in fossil fuels have resulted in renewed interest in the use of plants as direct sources of bioenergy and biomaterials (Henry, 2009a).

Humans domesticated plants more than 10 000 years ago largely for food (Fuller, 2007). Use of these species as energy crops risks conflict with food uses. Domesticating new species specifically for energy may allow access to species that are better suited to energy production and avoid diversion of food species. Accelerated domestication of these species should be able to take advantage of growing understanding of the process of domestication (Purugganan and Fuller, 2009) and knowledge of plant genomes based upon improved DNA analysis tools (Schuste, 2008). Production of biomaterials such as plastics (Somleva *et al.*, 2008) from plants requires the selection of appropriate species as production platforms (Simmons *et al.*, 2008). Transport energy in the form of biofuels requires sustainable high biomass production in a form that facilitates conversion to high quality fuel. The main risk associated with investment in the development of plants as biomass for bioenergy production is the possibility of advances in technologies for alternatives to liquid fuels such as electric cars, but this depends upon significant innovation in battery technology to match the energy density of liquid fuels (Tollefson, 2008a).

## First, second and later generation bioenergy from plants

The first generation of biofuel production has been based upon the conversion of the storage carbohydrates (sugars and starch) in the plants into fuel (Schubert, 2006). Oil from plants such as oilseeds has also been used, but the relatively low yields indicate that this is unlikely to be a sustainable source of fuel on a global basis. Engineering of improved oil composition (Graef *et al.*, 2009) may make these plants more suited to biodiesel production. However, the yield of oil per hectare from oil-producing plants will probably always be low compared to the yield per hectare of total biomass of the highest yielding plant that could be grown on the same land with the available water, nutrients and other inputs. The use of storage carbohydrates from the edible parts of plants creates the potential for direct competition between food and fuel production. The second generation of biofuels under development is based on the conversion of the structural carbohydrates of the plant cell wall (Yuan *et al.*, 2008). This avoids direct competition with food production and makes a much wider range of plants possible sources of biomass. Further developments are expected to allow conversion of plant biomass to higher value fuel molecules.

## Scale of plant production needed to replace fossil fuels

### How much land do we need to replace oil with crops?

A major question we need to ask is how much land do we need to grow plants to replace oil. The global consumption of oil in 2008 was around 3930 million tonnes. The area of land required can be calculated if we assume different levels of efficiency in converting plant biomass to biofuel (oil equivalent) at any given yield of biomass per hectare. Table 1 presents that the area required, in one extreme, is more than 3000 million ha at a crop yield of 5 tonnes per hectare and 25% conversion efficiency or, in the other extreme, around 100 million ha at 50 tonnes per hectare and 75% conversion efficiency. The need to produce food and other products from plants and the need to reserve land for biodiversity conservation suggest that the minimum possible land footprint would be desirable for energy production from plants.

**Table 1:** Estimates of area of land required to replace all oil with biofuel (based upon 3930 million tonnes/year fuel consumption as of 2008) (BP statistical review of world energy 2009, http://www.bp.com/productlanding.do?categoryId=6929&contentId=7044622)

Yield (tonnes/ha/year)	Proportion of biomass converted to biofuel (%)	Land area (Mha)
5	25	3142
	50	1571
	75	1047
10	25	1571
	50	786
	75	523
20	25	786
	50	393
	75	261
50	25	314
	50	157
	75	104

### What plants do we have with these yields?

The next question we need to address is what species of plants do we have with yields that would allow production on a reasonable land footprint. The yields of crops used as biomass sources need to be achievable on a sustainable basis with minimal energy inputs for crop production including cultivation, planting, nutrient production and application, harvesting and transport. This requirement limits the choice of crop species and production environments.

The biomass yield from plants varies enormously with environment. Consideration of yield potential under optimal conditions is a starting point. Grass species such as the major cereal crops provide yields of the order of 10 tonnes/ha/year of grain under favourable conditions. Total biomass yield potential is higher (around 20 tonnes/ha/year). Switchgrass (*Panicum virgatum*) has been widely evaluated as an energy crop option (McLaughlin *et al.*, 1999). Miscanthus has been shown to deliver much higher yields than currently available switchgrass genotypes (Boehmel *et al.*, 2008). Sugarcane and related species (*Saccharum, Miscanthus* and *Erianthus* species) are C4 plant with high yield potential. Sugarcane and related species are probably the grasses with the highest yield potential identified to date. Sugarcane has potential to yield in excess of 100 tonnes dry matter/ha/year (Bull and Glasziou, 1975). Maize and sorghum are potentially model genomes for research on the use of grasses as bioenergy crops (Carpita and McCann, 2008).

Woody biomass options include species such as poplar and willow with yield potentials of about 15 tonnes/ha/year (Strub *et al.*, 1983; El Bassam, 1998; Boehmel *et al.*, 2008). Eucalypt species have the potential to yield more than 100 tonnes/ha/year (Ugalde and Perez, 2001), comparable to the best grasses such as sugarcane. More than 700 Eucalypt taxa have been described with hybrids between these species displaying enhanced growth performance (Henry, 2009b). The poplar (Tuskan *et al.*, 2006) and Eucalypts are the emerging model genomes for woody plant development as bioenergy resources.

Many different plant species may be selected for energy production to suit the varied production environments available globally. As it matures, bioenergy production may parallel food production with many species being used at least regionally and a smaller number adapted to more diverse or abundant environments becoming widely grown internationally.

### What is the likely efficiency of conversion of plant biomass to biofuel?

Another key part of the requirement is that we have plants that can be converted at a high enough efficiency to ensure the land footprint is not too great. The conversion process is critical but the composition of the biomass can also be optimized to make the process more efficient. Selection of material with a high carbohydrate content and a composition that is easily degraded to sugars is best for current biochemical conversion technologies. Conversion efficiencies are low for most second-generation technologies but current research should provide significant advances (Simmons *et al.*, 2008).

### How much land is available for these crops?

The ultimate question is how much land is available for growing these plants. Most importantly, we need to consider the competition with land that may be used for food production or nature (biodiversity) conservation. The amount of arable land in the world has been estimated at more than 4000 Mha. The amount of land available by continent is given in Table 2. Much of this land is important for forests or biodiversity conservation and is not available for agriculture. The actual area of arable lands is much smaller. It may be possible to develop energy crops suitable for producing environments not currently considered to include arable land. However, the key issue is how much land would be required and the extent to which this would compete with other potential land uses. The use of high yielding sites may be attractive if as a consequence the land footprint can be small (Table 1). Estimation of the feasibility of production of any amount of biomass is complicated by the difficulty of defining the arable land. Much land that has not been classified as arable has been used for agriculture by adding water (irrigation) or nutrients (fertilizers) to otherwise infertile land. Species suited to production outside the range of environments used for major food crops may also increase the amount of land that might be available. The choice of species greatly influences the potential for production of biomass at any given location and a detailed analysis region by region is required for matching available land to available plant species.

**Table 2:** Arable land areas (http://www.fao.org/ag/agl/agll/terrastat)

Region	Potential arable land (Mha)
Asia and Pacific	778
Europe	384
North Africa and Near East	47
North America	480
North Asia	298
South and Central America	1028
Sub-Saharan Africa	1110
Total	4125

## Systematic analysis of plant genetic resources

Discovery of the best options for use as energy crops in specific environments requires an analysis of available species and their suitability in available production environments. A systematic analysis of plant options for food, energy, conservation and other uses should include all plant species. Those that do not have a current specific human use may have one in the future and all species contribute to biodiversity. Selection of new plants for energy production or even diversified food production requires a systematic analysis of the available options. Many of the species currently being promoted for use as energy crops have not been a product of such analysis and are in many cases not good options. For example, many oil-producing species are promoted because of the ease of using the oil produced in the plant with minimal processing. However, the environmental cost (land and water requirements) of growing these species will often not compete with many other species with much greater potential for biomass production. Application of appropriate selection criteria should focus attention on species with good potential for sustainable production. Following are some general criteria (Henry, 2009a) for use in selecting bioenergy crop species:

1. high suitability for genetic improvement
2. high biomass accumulation
3. high harvest index
4. high fraction of biofuel in harvested biomass
5. nutrients partition to nonharvested parts
6. being able to be grown on marginal lands
7. harvested material able to be stored in the field
8. high bulk density
9. high water use efficiency
10. high N use efficiency
11. low potential as a weed
12. high coproduct potential
13. optimal biomass composition
14. large-scale potential production
15. low cost of harvest

**Figure 1:**
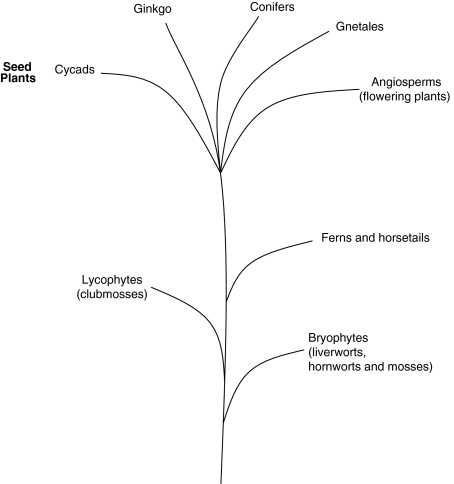
Relationships between higher plants. The relations of the five groups within the seed plants (Cycads, Gingo, Conifers, Gnetales and Flowering plants) have not been unambiguously resolved. The groups other than the seed plants have little human use for food, energy or other purposes.

Both algae and seed plants are actively being investigated as potential energy crops. The growth of higher plants is a well-established process, and the risks and likely outcomes of research in this area are more predictable. However, research aiming to develop technology to grow algae on a large scale is more uncertain but may produce a highly efficient energy production system. Analysis of specific plant families is a useful level for systematic analysis of options for plants and their current and potential uses. All plants in a family will not have the same utility, but they often share common biochemical features that make the family a useful level for analysis. Modern DNA analysis methods have greatly improved the rigour and utility of higher plant taxonomy. The variation in the composition of plants in relation to their utility can now be analysed against this taxonomy. For example, the distribution of the major components of plant biomass, the major structural and nonstructural carbohydrates in these families, deserves re-evaluation. A good example of how plant taxonomy and biochemical composition relate is found in the plant families reported to contain fructans (polymers of fructose that serve as reserve carbohydrates). Current DNA-based systematic analysis suggests many other related families that should be examined for the presence of fructans. The presence of nonstructural carbohydrates in the form of fructans has implications for food use and for energy production from these species. The type of structural carbohydrate (cell wall polysaccharides) can also be very important in determining the utility for food, feed or energy. The monocotyledonous plant families in the commelinoid group (including the grasses and related families) have cell walls rich in arabinoxylans and mixed linkage β-glucans (Henry and Harris, 1997). These cell walls are very different to those found in other higher plants and suggest very different processing requirements for conversion to biofuels. Comparative genomics allows the evolution of these different cell wall compositions to be followed (Fincher, 2009a,b;). The mixed linkage β-glucans appear to have evolved independently in the horsetails (Fry *et al.*, 2008). Very specific plant selection and improvement targets might be developed for improving groups of plants such as the grasses as resources for biofuels. Phylogenetic analysis is an important guide in our analysis of plant cell wall diversity and resulting potential for a wide range of uses. A descriptive list of families of seed-bearing plants is provided in Table S1. This provides a systematic analysis of the uses, current and potential, of higher plants family by family. Most plant families have not been adequately explored as bioenergy options. Conifers and related plants (Gymnosperms) are described by Hill (2005). Flowering plants (Angiosperms) are listed as defined by the Angiosperm Phylogeny Group (APG, 2003). Families that have been reported (Suzuki and Chatterton, 1993) to contain fructans are identified. The seed plants can be divided into five groups, four of which are gymnosperms, with the fifth being the flowering plants or angiosperms (Hill, 2005). The closest relatives to the seed plants are the ferns (Figure 1). The ferns and lower plants have limited food and other uses.

Many plants have been developed as food crops, and this suggests that we may need to domesticate many species to produce the biomass required to replace oil. Plants adapted to a wide range of available production environments are needed. The grasses (Poaceae) represent a major option having been domesticated for food production; they will probably repay screening efforts aiming to discover potential bioenergy crops. Many more plant families are probably able to contribute to the development of woody bioenergy tree crops. Some limited new options for domestication of food plants could also be identified by systematic analysis.

## Role of plant biotechnology

Plant biotechnology provides tools that may allow rapid development of domesticated genotypes with growth and composition characteristics optimized for energy production (Yuan *et al.*, 2008). Innovations that promote rapid biomass growth and development and engineering of cell wall biosynthetic pathways will be required. A high yield per unit of land area and a high conversion efficiency are essential to the delivery of an environmentally sustainable biofuel production system. Replacing oil with plant biomass probably needs to be associated with efforts to improve the efficiency of use of liquid fuels and reduce that total demand. It may be a priority to use plants to replace oil in the production of biomaterials other than fuel especially if other alternative renewable energy sources can be developed to replace transport energy requirements. Engineering plants to assist conversion to fuel by expressing enzymes required for processing (Taylor *et al.*, 2008) may make fuel production more efficient and economic. Developing plants to produce high value coproducts may also be necessary to provide an adequate economic return in production of biomass for energy. Plant biotechnology may contribute to the development of later generation biofuels that are more equivalent to current liquid fossil fuels such as gasoline or jet fuel (Tollefson, 2008b).

## Supporting information

Table S1 List of the available plant resources for food, fuel or other uses at the plant family level.
