# Supplementary material for: Evaluation of plant biomass resources available for replacement of fossil oil
Source: Plant Biotechnol J. 2010 Apr;8(3):288–93. doi: 10.1111/j.1467-7652.2009.00482.x (PMC2859252; doi:10.1111/j.1467-7652.2009.00482.x)
Supplement: Supplementary file 1 [file pbi0008-0288-SD1.doc]

**Table S1** List of the available plant resources for food, fuel or other uses at the plant family level

**GYMNOSPERMS**

The gymnosperms are the seed bearing plants that have exposed or uncovered ovules. From a human perspective these groups are second in importance only to the flowering plants. Hill (2005) provides an analysis of relationships within the gymnosperms. The Pinaceae within the Pinophyta is by far the most import economic group in the Gymnosperms.

CYCADAOPHYTA

The cycads are a distinct group of plants including 2 families (Cycadaceae and Zamiaceae), 10 genera and 300 species distributed in subtropical areas worldwide. The seeds and sometimes the stems of cycads have been eaten but require treatment to remove toxic compounds. Current uses are largely as ornamentals.

GINKGOPHYTA

This is a single species that is grown as an ornamental, *Ginkgo biloba*. This widely cultivated plant has edible seeds and has traditional medicinal uses.

PINOPYYTA

This group, known as the conifers, includes many important forest species in 7 families, 68 genera and 350 species. The conifers are widely disturbed but form dominant forests over large areas in the northern hemisphere. These trees are a major source of timber. This is the Gymnosperm family with greatest economic use and is probably the group with the greatest potential for development of further human uses.

**Auracariaceae** These are generally large trees with high value timber found in the southern hemisphere. Breeding of *Araucaria cunninghamii*, hoop pine has been in progress for many years. Crosses of geographically separated individuals result in improved growth presumably due to release from inbreeding within local populations of this wind pollinated species. *Araucaria bidwillii*, Bunya pine, and other Araucaria species produce an edible seed that has been a significant traditional food.

**Cephalotaxaceae** This family includes one genus with 5 species from the northern hemisphere.

**Cupressaceae** This family is found in both hemispheres and includes 30 genera. This family is also a significant source of timber.

**Pinaceae** These are 250 species from 10 genera found in the northern hemisphere. These are species are of great international importance as a source of timber. Selection and breeding for plantation production has included inter-specific hybridization. Edible pine nuts are produced from many species of pine that have seeds large enough to justify harvest. The large scale of production of these species suggests that these trees and even the waste from timber production in these species are important potential sources of energy. This is the most important group to humans within the Gymnosperms and remains the group with the greatest potential for exploration of additional future use.

**Podacarpaceae** This is another family found predominantly in the southern hemisphere with 18 genera and 180 species. This family is a source of high value timbers. The fruits of *Podocarpus elatus*, brown pine*,* have been eaten and have ongoing limited food use.

**Sciadopityaceae** This is a single species, *Sciadopitys verticillata*, from Japan.

**Taxaceae** This family contains 5 genera and 17 species mainly from the northern hemisphere. These species are also a significant source of timber. *Taxus brevifolia*, the pacific yew tree, is the source of paclitaxel, used to treat cancer.

GNETOPHYTA

This group includes 3 families (Gnetaceae, Ephedraceae and Welwitschiaceae), each with only one genus and 61 species found from tropical forests to deserts. These species are of little economic value. *Gnetum gnemon* from south east Asia and the Pacific has edible seeds.

**ANGIOSPERMS**

The angiosperms are the largest group of plants today with more than a quarter of a million species (AGPII, www.mobot.org/MOBOT/research/APweb/; Watson and Dallwitz, 1992). This group is of central importance to humans, accounting for most current use of plants by humans and most potential for future use.

Characteristically the Angiosperms have ovules enclosed in an ovary. An updated system of classification based upon DNA analysis, developed by the Angiosperm Phylogeny Group (APGII) has improved our understanding of the relationships within this most important group of plants (Henry, 2005). The families listed here include some that are optional (i.e. could either be considered separate families or part of a larger one). Chase (2005) provides an analysis of the relationships within the flowering plants.

The bulk of the angiosperms can be divided into the eudicots, monocots and the magnolids. However several small groups fall outside these groupings in molecular analysis of phylogenetic relationships in the angiosperms. These include the Amborellaceae (*Amborella* found only in New Caledonia) and the Nymphaceae (water lilies, minor food and ornamental uses). The Austrobaileyales (including Austrobaileyaceae, Schisandraceae, Illiciaceae and Trimeniaceae) are the next to split of from the main groups. Chinese star anise, *Illicium verum*, is used to flavour foods and is a member of the Iliciaceae. Next we need to consider the Ceratophyllaceae (monogeneric, used to protect fish in aquaculture) and the Chloranthaceae (4 genera, minor food and medicinal use, e.g. *Chloranthus officinalis* is used to make a tea). The position of these two groups has been difficult to determine. These so call ‘primitive’ angiosperms may have characters that are not especially primitive. Their phylogenetic position indicates their early divergence from other flowering plants found today. These groups do not currently have major human uses. They are a relatively small number of species worth conserving for their contribution to biodiversity and the evolutionary potential of flowering plants. They may be worth evaluating for genetic traits that could be useful to humans especially as whole genome analysis becomes a more routine technique.

MAGNOLIIDS

The Magnolids are a group of dicotyledonous plants more closely related to the monocotyledonous plants than to the other dicots (eudicots). Four orders may be defined: Canellales, Laurales, Magnoliales and Piperales. New uses in the future may include growth of some species as energy crops (eg Lauraceae).

 Canellales

The Canellales includes two small families, the Canellaceae (small trees from tropical Africa and America; includes *Canella winterana*, white cinnamon) and the Winteraceae (trees and shrubs from temperate forests of tropical areas especially southern hemisphere; includes *Drimys winteri*, winters bark, traditional medicinal use). Current attempts are being made to domesticate, *Tasmannia lanceolata*, mountain pepper, of eastern Australia as a food plant.

Laurales

The Laurales includes 7 families.

**Atherospermataceae** This small family of 5 genera and 12 species is found in the Australian region and in Chile. It includes aromatic species known as sassafras with limited use as timber.

**Calycanthaceae** This small family of shrubs from USA, east Asia and north Queensland, include *Calycanthus floridus*, Carolina allspice. Other species have limited use as perfumes and for traditional medicinal purposes.

**Gomortegaceae** This is a monospecific family from Chile. The single species, *Gomortega keule*, has no known uses.

**Hernandiaceae** The Hernandiaceae is a small tropical family with limited use as a source of timber.

**Lauraceae** The Lauraceae is a large family with more than 2000 species in over 30 genera from tropical and sub-tropical environments worldwide. Many species have valuable timber. *Persea americana*, avocado, is an important fruit. *Laurus nobilis*, bay laurel, is used to flavour foods. *Cinnamomum verum* is the source of cinnamon and *C. camphora* the source of camphor. These species may have potential as energy sources in some environments.

**Monimiaceae** The Monimiaceae is a medium sized family found in tropical areas and in the southern hemisphere.

**Siparunaceae** The family has two genera from tropical America and Africa with traditional medicinal uses.

Magnoliales

The Magnoliales includes 6 familes.

**Annonaceae** This large family of more than 2000 species is found throughout the tropics. Fruits include the *Annona* species, soursop, sweet sop and custard apple from south America. Others species are used as sources of perfumes and flavouring.

**Degeneriaceae** This family has single member, *Degeneria vitiensis*, from Fiji with no known uses.

**Eupomatiaceae** This family comprises two species *Eupomatia bennettii* and *E. laurina* from the rainforests of eastern Australia and New Guinea with very limited uses.

**Himantandraceae** This family has one genus, *Galbulimima* with three species from northern Australia and New Guinea with no known uses.

**Magnoliaceae** This is a small to medium sized family of ornamentals (especially the *Magnolia*) from Asia and America with valuable timbers and medicinal uses.

**Myristicaceae** The Myristiaceae is a medium sized family from throughout the tropics. The family includes *Myristica fragrans*, the source of nutmeg and mace. Other limited uses are as perfumes and waxes.

Piperales

The Piperales includes 5 families.

**Aristolochiaceae** This family of shrubs and herbs is found in tropical and temperate areas. A few are ornamentals (e.g. *Aristolocia macrophylla*, Dutchman’s pipe) and some have traditional medicinal uses.

**Hydnoraceae** This is a small family of parasitic leafless plants from Madagascar and Africa with no known uses.

**Lactoridaceae** This family comprises a single species of shrub, *Lactoris fernandeziana*, from the Juan Fernandez Islands in the south pacific with no known uses.

**Piperaceae** This large family has more than 2000 species distributed throughout the tropics.

*Piper nigrum*, pepper and *P. methysticum*, Kava are important economic species.

**Saururaceae** The Saururaceae is small family of perennial herbs from east Asia and north America with limited uses.

MONOCOTS

This group includes the current major food crops such as wheat and rice and current energy crops such as sugarcane and many potential energy and food crops of the future. The monocotyledonous plants can be divided into two groups the commelinids and the non-commelinids. These two groups differ in a number of key characteristics. The composition of the commelinid cell wall is distinct from that of the non-commelinids and other flowering plants. This is of great significance in relation to human uses as food or animal feed and may influence greatly our ability to digest or deconstruct the cell walls of these plants to release energy for biofuels.

The non-commelinid orders include the Acorales (1 family), Alismales (14 families), Asparagales (25 families), Dioscoreales (3 families), Liliales (10 families) and Pandanales (5 families),

**Petrosaviaceae** This family of non-commelinid monocots has not been placed in an order but includes 2 genera and 4 species of found at high elevations in east Asia with no known uses.

Acorales

**Acoraceae** The Acoraceae contains a single genus *Acorus* of 6 species from North America, south and east Asia with medicinal uses.

   Alismatales

The Alismatales includes 14 families.

**Alismataceae** This family of around 100 species is found in aquatic locations worldwide. The roots of *Sagittaria* species have been used as food.

**Aponogetonaceae** This small family has a single genus of aquatic plants, *Aponogeton*, with 45 species found in mainly tropical areas of Africa, south Asia and northern Australia. The tubers have been eaten.

**Araceae** This large family is widely distributed mainly in the tropics but also in temperate areas and has over 2000 species in more than 100 genera. The tubers of many species are a source of food. *Colocasia esculenta*, Taro, is an important starch crop. The *Monstera* inflorescence is also eaten. The arum lily is used as an ornamental.

**Butomaceae** This Butomaceae has a single species, *Butomus umbellatus*, or flowering rush from Europe and temperate Asia. This plant is cultivated as an ornamental and the rhizomes are sometimes eaten.

**Cymodoceaceae** The Cymodoceaceae is a small family (5 genera and 16 species) found in tropical and subtropical marine environments with indirect use as a source of habitat for important fish species.

**Hydrocharitaceae** This family of around 100 species is found in aquatic habitats worldwide and has been used as aquarium plants.

**Juncaginaceae**

This family of 3 genera and 14 species is found in aquatic environments worldwide in cold and temperate regions with some being edible.

**Limnocharitaceae** The Limnocharitaceae has about 12 species in 3 genera and is found in the tropics and sub-tropics in aquatic environments. *Limnocharis flava*, is used as a source of food and *Hydrocleis nymphoides*, water poppy, is a significant ornamental.

**Posidoniaceae** The Posidoniaceae has a single marine genus with 3 species found in the Mediterranean and along the southern Australian coast. The fibres are used to make fabrics.

**Potamogetonaceae** This family is found in aquatic habitats worldwide and includes around 100 species in two genera. These plants are important food sources for wild animals.

**Ruppiaceae** The Ruppiaceae has about 7 species in a single genus found worldwide mainly in areas between fresh and salt water with no known uses.

**Scheuchzeriaceae** This single genus, *Scheuchzeria*, has two species in cold areas of the northern hemisphere with no known uses.

**Tofieldiaceae** The Tofieldiaceae is a small family of about 6 species from several genera widely distributed in tropical and subtropical areas with little economic value.

**Zosteraceae** This family is found mainly in temperate marine environments and includes 3 genera and 18 species. These species are important food sources for fish species of economic importance.

  Asparagales

The Asparagales includes 24 families.

**Alliaceae** This family consists of 600 species in 13 genera including the important food species from the *Allium* genus, onion, shallot, garlic and leek.

**Agapanthaceae** The Agapanthaceae has a single genus, *Agapanthus*, with around 6 species from South Africa. Inter-specific hybrids are used as ornamentals.

**Amaryllidaceae** This large family of more than 1000 species is widespread in warm temperate and sub-tropical habitats. Important ornamentals include the *Narcissus sp.*, daffodils.

**Asparagaceae** The Asparagaceae is a widespread family with around 370 species in 1-3 genera. *Asparagus* *officinalis,* Asparagus is eaten as a vegetable.

**Agavaceae** This family of around 700 species and 30 genera is found in dry tropical and sub-tropical environments worldwide. Fibres (such as sisal hemp) are produced from *Agave* species. *Agave americana* is used to produce a Mexican drink. This family has been reported to store carbohydrates as fructans.

**Aphyllanthaceae** The Aphyllanthacea is represented by a single species, *Aphyllanthes monspeliensis*, from the western Mediterranean with no known uses.

**Hesperocallidaceae** The Hesperocalidaceae has single species, *Hesperocallis undulata,* desert lily, from south west North America with no known uses.

**Hyacinthaceae** The Hyacinthaceae contains around 70 genera and 1000 species from many areas especially South Africa and the Mediterranean. The family includes several ornamentals.

**Laxmanniaceae** The Laxmanniaceae consists of 15 genera and 180 species from Australasia, south east Asia and America. *Cordyline* species are used as ornamentals.

**Ruscaceae** The Ruscaceae has around 3 genera with 9 species from Africa and Europe including ornamentals such as *Dracaena*.

**Themidaceae** The Themidaceae is a family of 60 species in 12 genera found in north and central America with no known economic uses.

**Asteliaceae** The Asteliaceae includes around 30 species from 4 genera found in the southern hemisphere.

**Blandfordiaceae** This family includes the four species of the ornamental, *Blandifordia* species, Christmas bells, from eastern Australia. These species are now the subject of plant breeding.

**Boryaceae** The Boryaceae or lily trees are 12 species from 2 drought tolerant genera found in Australia.

**Doryanthaceae** This family is composed of two species of *Doryanthes* from central eastern Australia. They are cultivated as ornamentals. The roots of these plants are edible and the leaves are a source of fibre that was used traditionally for matting.

**Hypoxidaceae** The Hypoxidaceae is a widespread family including more than 100 species from 7 genera.

**Iridaceae** The Iridaceae is widely distributed in tropical and temperate regions with 70 genera and around 1750 species. This family includes ornamentals such as *Crocus, Freesia, Gladiolus and Iris*. These plants have been domesticated and subjected to considerable breeding. The food colouring saffron is obtained from the style of *Crocus sativus*. This family has been reported to store carbohydrates as fructans.

**Ixioliriaceae** The Ixioliriaceae includes single genus with a few species from south west Asia.

**Lanariaceae** This is a single species, *Lanaria lanata*, from South Africa

**Orchidaceae** This very large family has more than 20,000 species and includes many species cultivated as ornamentals that have been the subject of extensive breeding for ornamental values. The Orchidaceae and the Asteraceae are the two largest families of flowering plants. The popularity of many orchid species has resulted in excessive collection of wild populations increasing the risk of extinction in some cases. The flovouring, Vanillin, is obtained from *Vanilla planifolia.*

**Tecophilaeaceae**

This small family from Africa and America has no known uses.

**Xanthorrhoeaceae** This Xanthorrhoreaceae are a family of single genus *Xanthorrhoea* with 28 species from Australia, known as grass trees. The gum of grass trees is used in varnishes. This family has been reported to store carbohydrates as fructans.

**Asphodelaceae** The Asphodelaceae family includes around 850 species in 15 genera from sub-tropical and temperate Africa. Many species are ornamentals and the *Aloe* species are used for medicinal purposes.

**Hemerocallidaceae** This family includes 8 genera from Australasia, Europe and Asia. Ornamentals include the widely bred daylilies ([*Hemerocallis*](http://en.wikipedia.org/wiki/Hemerocallis)) and [New Zealand flax](http://en.wikipedia.org/wiki/New_Zealand_flax) ([*Phormium*](http://en.wikipedia.org/wiki/Phormium)), used as a source of fibre.

**Xeronemataceae**

This is a single genus, *Xeronema*, with one species from New Zealand and one from New Caledonia.

Dioscoreales

The Dioscoreales includes 3 families.

**Burmanniaceae** This family has about 12 genera and 100 species from tropical areas worldwide.

**Dioscoreaceae** This family of more than 600 species in 6 genera is found in the tropics and some temperate areas. *Dioscorea* species, yams, are cultivated as foods. These plants are also a source of a steroid.

**Nartheciaceae** This family has around 5 genera and 40 species.

Liliales

The Liliales includes 10 families.

**Alstroemeriaceae** The Alsteroemeriaceae has 4 genera and 200 species from central and South America. *Alsteroemerica* is an ornamental.

**Campynemataceae** This includes 2 genera with only a few species from Tasmanian and New Caledonia.

**Colchicaceae** The Colchicaceae has around 20 genera and 200 species from South Africa, Europe, central Asia and northern India including ornamentals.

**Corsiaceae** The Corsiaceae includes around 25 species from 3 genera. These are not autotrophic (not green) plants.

**Liliaceae** The Liliaceae has 16 genera and 600 species from the northern hemisphere. Ornamental species include *Lilium*, lilies and *Tulipa*, Tulips. This family has been reported to store carbohydrates as fructans.

**Luzuriagaceae** This family comprises 4 genera of tropical plants with few species.

**Melanthiaceae** The Melanthiaceae includes 3 genera with 18 species from southern Africa. Some have limited uses as timber, medicine or ornamentals.

**Philesiaceae** This family comprises a few species from 2 genera found in Chile.

**Rhipogonaceae** This single genus has 6 species from eastern Australia. *Rhipogonum scandens* is sometimes used for sarsaparilla. These plants are traditionally used for ropes.

**Smilacaceae** The Smilaceae is a family of 3 genera and around 370 species found in widely mainly in tropical and sub-tropical regions. *Smilax* species are used as source of sarsaparilla and some have traditional medicinal uses.

Pandanales

The Pandales includes 5 families.

**Cyclanthaceae** This family includes more than 200 species in 12 genera of tropical plants. Used for ornamental, medicinal purposes and weaving hats.

**Pandanaceae** The Pandaceae includes around 700 species in 3 genera from the tropical and subtropical regions of Africa, Asia and Australia. *Pandanus* (around 900 species) have edible starchy seeds. The leaves are used for weaving.

**Stemonaceae** The Stemonaceae are a small family from east and south east Asia, northern Australia and eastern North America. The family has limited uses with the roots of *Stemona* *tuberosa* having been used as a source of insecticide.

**Triuridaceae** The Triuridaceae is a family of 80 species and 9 genera of saprophytes from tropical and sub-tropical habitats in Africa, Asia and America.

**Velloziaceae** The Velloziaceae includes mainly woody shrubs from 6 genera and 270 species in tropical and sub-tropical areas and found in Africa, Madagascar, South America, China and Arabia. This family has no known uses.

COMMELINIDS

The commelinids are a distinct group of monocotyledonous plants.

**Dasypogonaceae** The Dasypogonaceae is a family of 4 genera and about 65 species of grass tree like plants from Australia, New Guinea and New Caledonia with no known uses.

Arecales

The Arecales has only one family the Arecaceae.

**Arecaceae** The Arecaceae, or palm family, comprises 190 genera and 2000 species, from mostly tropical environments. Food species include *Cocos nucifera*, coconut; *Phoenix* *dactylifera*, dates: *Metroxylon sp*., sago and *Elaeis guineensis*, palm oil. The fruits of other palms such as *Borassus flabellifera* , palmyra palm, are also edible. The Arecaceae are often used as ornamentals. Some species are used for ropes, coir and raffia.

Commelinales

The Commelinales has 5 families.

**Commelinaceae**

The Commelineaceae has 38 genera and more than 500 species found in tropical and warm areas worldwide. Ornamental members of this family include, *Commelina*, wandering Jew.

**Haemodoraceae** The Hemodoraceae includes 14 genera and 75 species. *Angiosanthos,* kangaroo paws, are cultivated as ornamentals. Breeding has produced hybrid ornamentals. This family has been reported to store carbohydrates as fructans.

**Hanguanaceae** The Hanguanaceae has one genus and 2 species.

**Philydraceae** The Philydraceae family includes 4 genera and 6 species.

**Pontederiaceae** The Pontederiaceae is composed of 10 genera and 30 species from fresh water habitats in the tropics worldwide. *Eichhornia crassipes*, water hyacinth, is a significant ornamental while some species are also important weeds.

Poales

The Poales is composed of 18 families.

**Anarthriaceae** The Anarthriaceae has 3 genera with about 12 species from south west Australia.

**Bromeliaceae** The Bromeliaceae includes 62 genera and 2900 species from mainly tropical parts of the Americas. *Ananas comosus*, the pineapple is an important fruit. The protein degrading enzyme, bromelain, is obtained from the pineapple plant. Some species are ornamentals.

**Centrolepidaceae** The Centrolepidaceae has 5 genera and 40 species from Australia, New Zealand, southern South America and south east Asia.

**Cyperaceae** The Cyperaceae has 120 genera and 500 species found worldwide in damp locations. The leaves are used for baskets and similar products.

**Ecdeiocoleaceae** The Ecdeiocoleaceae has a two species from south west Australia.

**Eriocaulaceae** The Eriocaulaceae is family of 10 genera and 1150 species from tropical and sub-tropical areas worldwide. They are used as ornamental, ‘everlastings’ when dried.

**Flagellariaceae** The Flagellariaceae is a single genus from tropical areas with several species in Africa south Asia and Australia. The stems are used for making baskets.

**Hydatellaceae** The Hydatellaceae includes 2 genera and 3 species of aquatic plants from Australia and India.

**Joinvilleaceae** The Joinvilleaceae has one genus with 2 species from Malaysia and the Pacific.

**Juncaceae** The Juncaceae has 80 genera and 100 species from wet areas worldwide. These species may be used for baskets and related products.

**Mayacaceae** The Mayaceae is single genus of water plants with 10 species from the Americas and west Africa. They are used in aquaria.

**Poaceae** The Poaceae is found worldwide and is of central importance to human societies. This large family includes the major cereal food crops (wheat, rice, maize, barley, sorghum, oats and millet) and these are the main human food crops. Cereal based food uses include a wide range of staples such as bread and rice and major beverages such as beer and whiskey. Another member of the family, sugarcane is probably the leading energy crop. Many other species have potential as energy crops and are being bred, selected or evaluated for energy use. Grasses are used widely as pasture or fodder for animals. Grasses are also bred for turf. Bamboo species are used for construction. This family will continue to be a resource for the identification and selection of plants for both new and traditional uses. Plant breeding in many of the major crop species in this group is a major public and private activity in many parts of the world. This family has been reported to store carbohydrates as fructans. The family is a major source of species with potential as new food and energy crops. Species such as Miscanthus and switchgrass are currently being evaluated but many more energy crops are likely to be found in the 10,000 or more species in this family. New food species are also likely to be developed by systematic analysis and development within this family.

**Rapateaceae** The Rapateaceae has 17 genera and 80 species tropical areas in South America and west Africa. They have no known uses.

**Restionaceae** The Restionaceae includes 35 genera and 320 species from the Southern Hemisphere mainly in southern Africa and Australia.

**Sparganiaceae** The Sparganiaceae has a single genus, *Sparganium*, with 20 species that may be part of the Typhaceae.

**Thurniaceae** The Thurniaceae includes a genus with 3 species found in moist locations in north east Brazil and Guiana. These species have no known uses.

**Typhaceae** The Typhaceae consists of the single genus of bulrushes, Typha, with about 10 species found in fresh water worldwide. The rhizome of these species may be eaten.

**Xyridaceae** The Xyridaceae contains 5 genera and 270 species from wet areas of the tropics. Some species have medicinal uses or are used in aquaria.

Zingiberales

The Zingiberales includes 8 families.

**Cannaceae** The Cannaceae has single genus of 55 species from central America. The *Cannas* are ornamentals. The starch of Queensland arrowroot, *Canna edulis* is used as food.

**Costaceae** The Costaceae are found in tropical areas and includes 4 genera and around 200 species.

**Heliconiaceae** The Heliconiaceae is a single genus, *Heliconia*, from the Americas and pacific with more than 100 species. Many ornamentals have been bred.

**Lowiaceae** The Lowiaceae is single genus with 2 species from China and south Asia.

**Marantaceae** The Marantacea has 30 genera and 400 species from tropical areas. Arrowroot, *Maranta arundinaceae*, is a source of starch for foods. The flowers of two species, *Calathea macrosepala* and *C. violacea* from Mexico are eaten.

**Musaceae** The Musaceae includes 3 genera and 42 species from Africa south Asia and the Pacific. The cultivated banana is a major food crop derived from *Musa acuminata* and *M. balbisiana*.

**Strelitziaceae** The Strelitziaceae has 3 genera and 7 species from tropical areas including significant ornamentals.

**Zingiberaceae** The Zingerberaceae is a family of 45 genera and 700 species. *Zingiber officinale*, ginger, is used as a food and food flavouring. *Curcuma,* tumeric, and *Amomum*, cardamom, are also important food flavourings. Some species are used as ornamentals.

EUDICOTS

The Eudicots are a large and distinct group of dicotyledonous plants with almost 200,000 species. This group includes many of the species of higher plants and many of the opportunities for discovery of new food and energy plants.

**Buxaceae** The Buxaceae or box family is widespread with 4 genera and around 100 species. The trees are important ornamentals. Some are used as hedges and some have valuable timber.

**Didymelaceae** The Didymelaceae is single genus with two species from Madagascar and Comoro Island.

**Sabiaceae** The Sabiaceae has one genus and 55 species of mainly climbers from tropical and warm areas of America and Asia.

**Trochodendraceae** This family is a single species from Japan.

**Tetracentraceae** The Tetracentraceae includes a single species from south Asia, India and China.

Proteales

The Proteales contains 3 families

**Nelumbonaceae** The Nelumbonaceae has a single genus with 2 species found in fresh water in Asia and Australia. The tubers, leaves and seeds of *Nelumbo nucifer*, sacred lotus, are used as a food

**Proteaceae** The Proteaceae is a family of more than 80 genera and 1500 species mainly from seasonally dry regions in the southern Hemisphere. Many species are ornamentals and have been subjected to some breeding. *Macadamia* species have an edible nut that is cultivated and has been subjected to limited breeding. Some rainforest species have valuable timber. The largest genus, *Grevillea*, has more than 350 species and has been the subject of breeding producing ornamental hybrids.

**Platanaceae** Plantanaceae is the plane tree family from the northern Hemisphere with one genus and 10 species. These trees are widely planted worldwide.

Ranunculales

The Ranunculales has 7 families.

**Berberidaceae** The Berberidaceae or barberry family contains 15 genera and 570 species that are widespread in temperate and sub-tropical environments of the northern Hemisphere and South America. *Berberis* sp., barberry and *Mahonia aquifolium*, Oregon grape, are edible species.

**Circaeasteraceae** This is a single species *Circaester* *agrestis*, from the Himalayas and south west China.

**Kingdoniaceae** This is a single species, *Kingdonia uniflora*, from north and west China.

**Eupteleaceae** The Euptelaceae has single genus, *Euptelea*, with 2 species from East Asia.

**Lardizabalaceae** The Lardizabalaceae has 8 genera and 35 species from east Asia and South America. Some have edible fruits.

**Menispermaceae** The Menispermaceae is a family of mainly climbing plants from East Asia and North America. It includes 75 genera and 520 species.

**Papaveraceae** The Papaveraceae or poppy family includes 26 genera and 250 species. Opium is obtained from *Papaver somniferum* and many species are ornamentals.

**Fumariaceae** The Fumariaceae includes 15 genera and 450 species from the Northern hemisphere and South Africa.

**Pteridophyllaceae** This is a single species, *Pteridophyllum racemosum*, from Japan.

**Ranunculaceae** The Ranunculace or buttercup family is widespread with about 50 genera and 1500 species. Many are ornamentals and some have medicinal uses. The seeds of [*Nigella sativa*](http://en.wikipedia.org/wiki/Nigella_sativa) are used as a spice.

 CORE EUDICOTS

The Core Eudicots defined in the APGII system includes 4 or more orders in addition to the more than 20 in the Rosids and Asterids

**Aextoxicaceae** This is a single species**,** *Aextoxicon punctatum,* a tree from Chile.

**Berberidopsidaceae** This family has 2 genera and 3 species.

**Dilleniaceae** The Dilleniaceae has 11 genera and 2400 species and is widespread in temperate, subtropical and tropical areas and especially Australia. *Hibbertia* species are grown as ornamentals.

Gunnerales

The Gunnerales includes 2 families.

**Gunneraceae** The Gunneraceae has a single genus, *Gunnera*, with 50 species from temperate to tropical environments.

**Myrothamnaceae** This family has one genus with 2 species from tropical Africa.

Caryophyllales

The Carophyllales includes 29 families.

**Achatocarpaceae** The Achatocarpaceae has 2 genera with 10 species from warm to tropical areas of the USA and South America.

**Aizoaceae** The Aizoaceae includes 135 genera and 1900 species from arid areas of Southern Africa, Australia and the Pacific. The fruits of the ice plant or pigface, *Carpobrotus edulis* are edible. The leaves of New Zealand spinach, *Tetragonia tetragonoides* are eaten as a vegetable.

**Amaranthaceae** The Amaranthaceae has 160 genera and 2400 species from cool and temperate regions. The family includes ornamentals and the seeds of *Amaranthus* are eaten.

**Ancistrocladaceae** This includes single genus of vines with 12 species found in tropical areas from Africa to Malayasia.

**Asteropeiaceae** The family has single genus with 7 species from Madagascar.

**Barbeuiaceae** This family is a single species from Madagascar.

**Basellaceae** The Basellaceae includes 4 genera and 25 species from tropical America, Africa and Asia. *Ullucus tuberosus* is grown as a major root vegetable in South America. *Basella alba* is used in Asian food.

**Cactaceae** The Cataceae or cactus family of 97 genera and 1400 species is found in the Americas. *Opuntia* species, prickly pear and *Hylocereus* species have fruits that are eaten. Many species are grown as ornamentals.

**Caryophyllaceae** The Caryophyllaceae or carnation family has around 87 genera and 2300 species. This widely distributed family from mainly temperate areas includes many ornamentals.

**Didiereaceae** This family includes 4 genera and 11 species from Madagascar.

**Dioncophyllaceae** This is a family of vines from west Africa including 3 species in 3 genera.

**Droseraceae** The Droseraceae, sundew family is a widespread family of carnivorous plants from 4 genera and 105 species.

**Drosophyllaceae** This is a single species, the Portuguese sundew, *Drosophyllum lusitanicum.*

**Frankeniaceae** Frankeniaceae is widespread with 4 genera and 90 species.

**Gisekiaceae** This is a single genus with 7 species from tropical Africa.

**Halophytaceae** This is a single species from Patagonia.

**Molluginaceae** The Molluginaceae is widespread and has 15 genera and 100 species.

**Nepenthaceae** The Nepenthaceae, tropical pitcher plant family, has 2 genera and 68 species from south east Asia, northern Australia and Madagascar. These are highly valued ornamentals.

**Nyctaginaceae** The Nyctaginaceae, four o’clock family, is widely distributed with 33 genera and 290 species. The family includes ornamentals such as *Mirabilis* and *Bougainvillea.*

**Physenaceae** This family is a genus of 2 species from Madagascar.

**Phytolaccaceae** The pokeweed family is widespread with 15 genera and 100 species.

**Plumbaginaceae** The Plumbaginaceae or plumbago family is a widespread family of 24 genera and 775 species with some ornamentals.

**Polygonaceae** The Polygonaceae or knotweed family is widespread with more than 40 genera and more than 1000 species.

**Portulacaceae** The Portulaceae is widespread and has 20 genera and 580 species. Some species are ornamentals and *Portulaca oleracea*, purslane, is edible.

**Rhabdodendraceae** This is a genus with 4 species from Brazil and the Guianas.

**Sarcobataceae** Sarobataceae has a single species of halophyte from North America.

**Simmondsiaceae** The jojoba family is a single species, *Simmondsia chinensis*, from California. This plant is grown for the oil or wax.

**Stegnospermataceae** This family has a single genus with 3 species from central America.

**Tamaricaceae** The Tamaricaceae has 4 genera with 120 species from temperate and sub-tropical areas worldwide except the Americas.

Santalales

The Santalales includes 5 families.

**Olacaceae** The Olaceae includes 25 genera and 250 species of plants from the tropics with some medicinal uses.

**Opiliaceae** The Opiliaceae includes 10 genera and 28 tropical species.

**Loranthaceae** The Loranthaceae, a family of mistletoe, has 70 genera and 940 species worldwide in tropical and warm environments especially in the south. These plants are partly parasitic on other plants.

**Misodendraceae** This is a genus of parasitic plants with 11 species from temperate South America.

**Santalaceae** The Santalaceae or sandalwood family includes 38 genera and 400 species of partly parasitic plants from warm areas worldwide. Some species have edible fruit. They are also sources of valuable timber used for perfume. Breeding and selection in this group has been very limited.

Saxifragales

The Saxifragales includes 14 families.

**Altingiaceae** The Altingiaceae is a small family widespread in temperate to tropical areas with 3 genera and 10 species. These trees are a source of timber and are planted as ornamentals (eg *Liquidambar*).

**Aphanopetalaceae** This family is represented by 2 species of vine like shrubs from Australia.

**Cercidiphyllaceae** This family comprises 2 ornamental tree species from China and Japan.

**Crassulaceae** The Crassulaceae or orpine family is very widespread with 40 genera and 1500 species including ornamental species. A pathway of photosynthesis, the Crassulacean Acid Metabolism or CAM pathway is named after this group where it was first discovered.

**Daphniphyllaceae** This is a single genus of 35 species found in East Asia.

**Grossulariaceae** This family includes only the genus, *Ribes,* with around 150 species including currants, gooseberries and ornamentals and medicinal plants.

**Haloragaceae** This family of 9 genera and 145 species is found worldwide but especially in Australia and the Southern hemisphere.

**Penthoraceae**

This family has single genus, *Penthorum,* with 2 species from North America and East Asia.

**Tetracarpaeaceae** This family is single species, *Tetracarpaea tasmanniaca* from Tasmania.

**Hamamelidaceae** The Hamamelidaceaeis a widespread mostly sub-tropical family including 27 genera and 90 species.

**Iteaceae** The Iteaceae has 2 genera with 17 species of temperate to tropical areas of Asia, North America and South Africa.

**Pterostemonaceae** This family is a single genus with 2 species from Mexico.

**Paeoniaceae** *Paeonia* is the only genus with 30 species including ornamentals (Peony) from Asia, Europe and North America.

**Saxifragaceae** The Saxifragaceae includes 36 genera and 460 species with some ornamentals and is largely from northern temperate environments.

ROSIDS

The Rosids are a very large group of 13-16 orders.

**Aphloiaceae** This is a single species, *Aphloia theiformis*, from east Africa and the Indian Ocean.

**Geissolomataceae** This family has a single species, *Geissoloma marginatum*, from Southern Africa.

**Ixerbaceae** The Ixerbacea is a family of one species, *Ixerba brexioides*, from New Zealand.

**Picramniaceae** The Picramniaceae has 2 genera and 50 species from tropical and central America.

**Strasburgeriaceae** This family has one species, *Strasburgia calliantha*, from New Caledonia.

**Vitaceae** The Vitaceae has 15 genera and 700 species from a wide range of temperate to tropical environments. The grape, *Vitis vinerifera*, has been widely cultivated as fruit and to produce wine. The many wild species provide an important genetic resource for grape breeding into the future.

Crossosomatales

Crossosomataceae

The Crossosomataceae includes 3 genera and 10 species from North America.

**Stachyuraceae**

This family has a single genus of 10 species from Asia.

**Staphyleaceae**

The Staphyleaceae has 5 genera and 60 species from the northern hemisphere and South America including some ornamentals.

Geraniales

The Geraniales includes 6 families.

**Geraniaceae** The Geraniaceae, Geranium family, is widely distributed in mainly temperate and sub-tropical areas and has 11 genera and 700 species including the ornamental Geraniums and Pelargoniums. The oil is used in perfume.

**Hypseocharitaceae** This family has one genus of 8 species from the Andes.

**Ledocarpaceae** The Lepocarpaceae has 2 genera and 11 species from the Andes.

**Melianthaceae** The Meliantahceae has 2 genera and 15 species from Africa.

**Francoaceae** This family has 2 genera and 2 species from Chile including the Bridal Wreath.

**Vivianiaceae** This family consists of 2 genera and 30 species from South America.

 Myrtales

The Myrtales includes 14 families.

**Alzateaceae** This is a single species, *Alztea verticillata*, from tropical areas of central and South America.

**Combretaceae** The Combretaceae is a widespread family from tropical and sub-tropical regions and has 20 genera and 600 species. Some species are used for timber.

**Crypteroniaceae** This family includes 3 genera and 10 species from tropical Asia.

**Heteropyxidaceae** This is a single genus with 3 species from South Africa.

**Lythraceae** The Lythraceae is widespread including 28 genera and 580 species. This family includes, *Punica granatum*, the pomegranate.

**Melastomataceae** The Melastromaceae includes 188 genera and 4,950 species from tropical and sub-tropical environments. Some species are used as ornamentals, for timber or have edible fruits.

**Memecylaceae** The Memecylaceae has 7 genera and 430 species from tropical areas.

**Myrtaceae** The Myrtaceae or myrtle family is predominantly found in tropical and sub-tropical areas and in the southern hemisphere. The family is a source of timber (Eucalypts) and essential oils and includes plants with edible fruits (eg *Syzygium* species). *Psidium guajava*, guava originates from tropical America. *Syzgium aromaticum*, cloves and *Pimenta dioica*, allspice are also members of the Myrtaceae. The Eucalypts perform well in a wide range of marginal environments making them important energy crop options.

**Oliniaceae** This is a single genus with 10 species from Africa.

**Onagraceae** The Onagraceae or evening primrose family has 24 genera and 650 species with a wide distribution and includes ornamentals such as *Fuchsia*.

**Penaeaceae** The Penaeaceae includes 7 genera and 25 species from South Africa.

**Psiloxylaceae** This is a single species from the Mascarene Islands.

**Rhynchocalycaceae** This is a single species from South Africa.

**Vochysiaceae** The Vochysiaceae includes 7 genera and 200 species from tropical areas.

EUROSIDS I

The Eurosid I group includes 7-8 orders.

**Zygophyllacea** This family is widespread in the tropics with 30 genera and 235 species. The dense wood, lignum vitae, is produced from species of *Guaiacum*. Some species are ornamentals and some have medicinal uses.

**Krameriaceae** The Krameriaceae has a single widespread genus with 18 species that includes partial parasites and plants with medicinal use.

**Huaceae** The Huaceae has 2 genera and 3 species from the tropics.

  Celastrales

**Celastraceae** This is a family of mainly tropical and sometimes climbing plants with around 100 genera and 1000 species.

**Lepidobotryaceae** This family is a single species from tropical Africa and America.

**Parnassiaceae** This is a single genus of northern temperate plants including 50 species.

**Lepuropetalaceae** This is single American species, *Lepuropetalon spathulatum*.

Cucurbitales

The Cucurbitales consists of 7 families.

**Anisophylleaceae** This is a widespread tropical family with 4 genera with 36 species.

**Begoniaceae** The Begonia (ornamentals) family has around 3 genera and 950 species from tropical areas worldwide.

**Coriariaceae** This is a single genus, *Coriaria*, with 15 highly toxic species some of which are cultivated as ornamentals.

**Corynocarpaceae** This single genus, *Corynocarpus*, has 5 species distributed from Malaysia to New Zealand.

**Cucurbitaceae** The Curcurbitaceae has 120 genera and 640 species from warm to tropical areas worldwide. Important food species include; pumpkins, squashes, gourds and marrows, *Cucurbita* species; melon, cantaloupe, honeydew and cucumber, *Cucumis* species and watermelon, *Citrullus lanatus. Lagenaria leucantha*, bottle gourd is a very widely and long cultivated vegetable.

**Datiscaceae** Datiscaceae has 2 species of *Datisca* from dry areas of Europe, Asia and North America.

**Tetramelaceae** The Tetramelaceae includes 2 genera and 2 species from tropical south east Asia and Australia.

  Fabales

The Fabales includes 4 families.

**Fabaceae**

The Fabaceae or legume family is the third largest family of flowering plants with 720 genera and 19, 400 species. The family includes many important food crops such as [*Glycine max*](http://en.wikipedia.org/wiki/Glycine_max), soya bean, [*Phaseolus*](http://en.wikipedia.org/wiki/Phaseolus)*,* various types of bean, [*Pisum sativum*](http://en.wikipedia.org/wiki/Pisum_sativum), pea , [*Medicago sativa*](http://en.wikipedia.org/wiki/Medicago_sativa), alfalfa, and [*Arachis hypogaea*](http://en.wikipedia.org/wiki/Arachis_hypogaea) , peanut. These species are the subject of considerable breeding effort. These plants are an important source of protein in human doest especially for vegetarians.

**Polygalaceae** The Polygalaceae, or milkwort family, are a widespread group with 17 genera and 800 species.

**Quillajaceae** This is a single genus with 2 species from warm temperate South America.

**Surianaceae** This family includes 4 genera and 5 species from tropical and sub-tropical regions.

Fagales

The Fagales has 7 families.

**Betulaceae** The Betulaceae or birch family has 6 genera and 120 species. *Corylus avellana* is the source of the hazel nut. The family also includes ornamental and timber species.

**Casuarinaceae** The Casuarinaceae or she-oak family has 4 genera and 65 species from Australia, Malaysia, New Caledonia and Fiji. These species are used for their timber and as ornamentals. The roots have nitrogen-fixing nodules associated.

**Fagaceae** The Fagaceae or beech family has 9 genera and about 900 species from the northern hemisphere. Some species provide timber or are ornamentals. Cork is produced from the bark of *Quercus suber. Castanea* species are the source of chestnuts*.*

**Juglandaceae** The Juglandaceae or walnut family has 8 genera and 50 species found in Europe, Asia, America and south east Asia. *Juglans regina*, walnut and *Carya ilinoinensis*, pecan are important nut crops. Valuable timbers are also obtained from this family.

**Rhoipteleaceae** This is a single species, *Rhoiptelea chiliantha*, the horsetail tree from the mountains of China and Vietnam.

**Myricaceae** The Myricaceae has 4 genera and 40 species. Wax and edible fruits are produced from species of this family.

**Nothofagaceae** The Nothofagaceae or southern beech family includes 35 species of the genus, *Nothofagus*, found in the southern hemisphere.

**Ticodendraceae** This family is comprised of a single species, *Ticodendron incognitum*, from central America.

 Malpighiales

The Malpighiales includes 29 families. Details are provides here for 13 examples.

**Achariaceae** This family has 3 genera and 3 species from South Africa.

**Clusiaceae** This is a widespread family of 40 genera and 1000 species. *Garcinia mangostana*, mangosteen and *Mammea americana*, mamme apple are sources of fruit.

**Balanopaceae** The Balanopaceae is single genus of 12 species from Queensland, New Caledonia and Fiji. These species have no known uses.

**Bonnetiaceae** This is a family of 4 genera and 32 species from the tropics.

**Caryocaraceae** This tropical family includes 2 genera and 25 species.

**Chrysobalanaceae** This family has 17 genera and 400 species widespread in the tropics.

**Euphorbiaceae** The Euphorbiaceae or spurge family is a very large family with 300 genera and around 5000 species found mostly in tropical but also some temperate regions. *Heva brasiliensis* is the source of rubber. *Manihot esculenta*, cassava is an important staple food. *Ricinus communis* is the plant used to produce Castor oil. *Mallotus philippinesis* produces a red dye. Ornamental species from the family include poinsettia.

**Linaceae** The widespread Linaceae has 8 genera and 90 species. Flax is obtained from *Linum usitatissimum.*

**Pandaceae** The Pandaceae includes 4 genera and 28 species from Africa and south east Asia.

**Passifloraceae** This family has 18 genera and 530 species from tropical and sub-tropical areas. *Passiflora* species produce the edible passion fruit and some species are ornamentals.

**Phyllanthaceae** The Phyllanthaceae comprises 56 genera and 1700 species and is widespread in the tropics. The fruits of *Baccaurea* species, tampoi trees are edible. *Cicca acida*, Malay gooseberry is edible.

**Salicaceae** The Saliaceae has 4 genera with 350 species and are found worldwide except Australia. *Populus* or poplar and *Salix* or willow is used as source of wood and may become important energy crops.

**Violaceae** The Violaceae has 21 genera and 900 species found worldwide and used as ornamentals.

Oxalidales

The Oxalidales contains 6 families.

**Brunelliaceae** This family has a single genus, *Brunellia*, with 45 species from tropical America.

**Cephalotaceae** This is a single species, *Cephalotus follicularis*, the flycatcher plant from southwest Australia. These carnivorous plants are grown as ornamentals.

**Connaraceae** The Connaraceae includes 16 genera and 350 species from the tropics. The family includes some medicinal species. *Connarus guianesis* from Guyana is used as a source of zebra wood.

**Cunoniaceae** The Cunoniaceae has 26 genera and 350 species from Australia, New Zealand, New Caledonia, New Guinea, and South America. *Ceratopetalum apetalum* and other species are used as source of timber. The *Davidsonia* species have edible fruits.

**Elaeocarpaceae** The Elaeocarpaceae has 12 genera with 605 species found in Madagascar, south east Asia, Australia, New Zealand, Chile and the Caribbean. Some species have limited ornamental and medicinal uses and some (*Elaeocarpus* species) have edible fruits. *Elaeocarpus grandis* is used for timber.

**Oxalidaceae** The Oxalidaceae has 3 genera and 875 species mainly from the tropics and sub-tropics. The tubers and leaves of some *Oxalis* species are eaten. *Averrhoa carambola*, carambola (starfruit) and *Averrhoa bilimbi,* bilimbi are species from southern Asia. Some species are difficult weeds.

  Rosales

The Rosales has 9 families.

**Barbeyaceae** This family is a single species, *Barbeya oleoides*, from north east Africa and Arabia.

**Cannabaceae** The Cannabaceae has 2 genera and 3 species . Economic species include *Cannabis sativa*, hemp used for fibre, oil and medicinal applications (and as an intoxicant as marijuana) and *Humulus lupulus,* hops, used to flavour beer.

**Dirachmaceae** This family has single genus with two species from east Africa.

**Elaeagnaceae** The Elaeaganaceae has 3 genera and 50 species from mainly northern temperate environments but also from eastern Australia. The family includes some species with edible fruit.

**Moraceae** The Moraceae or fig family has 40 genera and 1000 species from tropical and sub-tropical regions. Economic species include the *Ficus carica*, fig, *Morus* species, mulberry, *Artocarpus* *altilis*, breadfruit, *Artocarpus heterophyllus*, jackfruit and *Ficus* *benghalensis*, banyan.

**Rhamnaceae** The Rhamnaceae has 900 species and 50 genera found worldwide and including some species with edible fruits. The family also includes some timber, medicinal and ornamental species.

**Rosaceae** The Rosaceae or rose family has more than 100 genera and 3000 species found worldwide. Important food species include; *Malus,* apples; *Pyrus*, pears; *Prunus*, cherries, plums, nectarines, peaches, apricots and almonds; *Rubus*, raspberries, loganberries, blackberries; *Eriobotrya*, loquats; *Fragaria*, strawberry and *Cydonia*, quinces. Many of these are now the subject of intensive plant breeding programs. Roses are highly valued and bred ornamentals. The Rosaceae store some of their carbohydrates as the sugar alcohol, glucitiol (sorbitol). The many important food and ornamental species in this family may have potential as a source of biomass for energy at least for non food component of the biomass.

**Ulmaceae** The Ulmaceae has 15 genera and 200 species found worldwide including some timber species.

**Urticaceae** The Urticaceae is found worldwide and includes 49 genera and 550 species. Some nettles (*Urtica* species) are eaten. *Boehmeria nivea*, ramie is used for fibre for the production of cloth.

EUROSIDS II

The Eurosids II includes 3-4 orders.

**Tapisciaceae** This family has 2 genera from China and the Caribbean.

Brassicales

The Brassicales includes 15-17 families.

**Akaniaceae** This family is a single species, *Akania licens*, Turnipwood, from eastern Australia.

**Bretschneideraceae** This is a single genus with 2 species from China.

**Bataceae** This is a single genus, *Batis*, with 2 salt tolerant species from tropical America and Australia.

**Brassicaceae** The Brassicaceae is a large family of 330 genera and 3700 species found worldwide. [*Brassica oleracea*](http://en.wikipedia.org/wiki/Brassica_oleracea) , cabbage, cauliflower, Brussel sprout, broccoli and [*Brassica campestris*](http://en.wikipedia.org/wiki/Brassica_rapa), turnip, and Chinese cabbage are important vegetables. Oilseeds are produced from [*Brassica campertris and B. napus*](http://en.wikipedia.org/wiki/Brassica_napus) (rapeseed). [*Raphanus sativus*](http://en.wikipedia.org/wiki/Raphanus_sativus), radish and [*Armoracia rusticana*](http://en.wikipedia.org/wiki/Armoracia_rusticana), horseradish are also significant foods. *Wasabi japonica* is the source of Japanese wasabi. *Arabidopsis thaliana* has been used as model plant to study flowering plants.

**Caricaceae** The Caricaceae has 4 genera and 55 species from tropical and sub-tropical America and west Africa. *Carica papaya*, papaw or papaya is an important fruit.

**Emblingiaceae** This is a single species, *Embilginia calceoliflora,* from Western Australia.

**Gyrostemonaceae** The Gyrostemonaceae has 5 genera and 16 species from Australia.

**Koeberliniaceae** This is a single species *Koeberlinia spinosa*, from USA and Mexico.

**Limnanthaceae** This family has 2 genera and 11 species from temperate North America.

**Moringaceae** This is a single genus with 12 species. *Moringa oleifera*, horseradish tree, is used for food with edible fruits and roots and the seed are a source of perfume and lubricant.

**Pentadiplandraceae** This is a genus, *Pentadiplandra*, with 2 species from tropical Africa.

**Resedaceae** The Resedaceae has 6 genera and 70 species from temperate to sub-tropical areas. This family includes species used as medicines, dyes and perfume.

**Salvadoraceae** This family has 3 genera and 12 species from sub-tropical and tropical Africa and southern Asia.

**Setchellanthaceae** This family comprises the species, *Setchellanthus caeruleus.*

**Tovariaceae** This is a single genus, *Tovaria*, with 2 species central and South America and the West Indies.

**Tropaeolaceae** The Tropaeolaceae has 3 genera and 92 species from temperate to tropical areas of Mexico and South America. *Tropaeolum* species, Nasturtiums, have edible flowers.

Malvales

The Malvales has 10 families.

**Bixaceae** The Bixaceae has single genus with 4 species from the tropic and sub-tropics of America and the Caribbean. *Bixa* *orellana* is used a source of red food colouring and flavourings and as dye for cosmetics.

**Diegodendraceae** This is a single species, *Diegodendron humbertii* , from Madagascar.

**Cochlospermaceae** The Cochlospermaceae has 2 genera and 25 species from the tropics. The species have limited ornamental and medicinal uses and one is used for cordage.

**Cistaceae** The Cistaceae or rock rose family has 9 genera and 200 species mainly from Europe and North America with ornamental uses. Perfume is also sourced from some species.

**Malvaceae** The Malvaceae has 100 genera and 1000 species found worldwide. *Gossypium* species, cotton and *Hibiscus cannabinus*, kanaf are important fibre crops. *Hibiscus* species are used as ornamentals. *Sterculia* species, tropical chestnuts are edible. *Durio zibethinus*, durian, is a fruit of controversial flavour. Other species from this genus are also edible.

**Muntingiaceae** This family has 3 genera and 3 species from the tropics. Fruit of the ornamental, *Muntigia calabura,* are edible**.**

**Neuradaceae** The Neuradaceae has 3 genera and 10 species distributed from the Mediterranean to India.

**Sarcolaenaceae** This family has 10 genera and 40 species from Madagascar.

**Sphaerosepalaceae** This family has 2 genera and 14 species from Madagascar.

**Thymelaeaceae** The Thymelaeaceae is very widespread and has 44 genera and 500 species mostly in the southern hemisphere. The family includes many ornamentals. Some species have provided a source of fibre. *Pimelea* bark has been used as twine. *Gonystylus* species from south east Asia produce a high value timber.

Sapindales

The Sapindales consist of 11 families.

**Anacardiaceae** The Anacardiaceae or cashew family includes 70 genera and 600 species and is widespread in the tropics. *Mangifera indica*, (and other *Mangifera* species) mango, *Anacardium occidentale*, cashew nut, *Pistacia vera*, pistachio nut are important food species. *Spondias cytherea*, golden apple fruits are edible. *Toxicodendron radicans,* poison ivy is well known toxic plant in this family.

**Biebersteiniaceae** This is a single genus with 5 species found from south west Europe to central Asia.

**Burseraceae** The Burseraceae includes 17 genera and 500 species that are widespread in tropical and sub-tropical areas. The family includes ornamentals and timber species. *Boswellia carterii* is the source of frankincense used in incense and perfumes.

**Kirkiaceae** The Kirkiaceae is a single genus with 8 species from tropical and south Africa.

**Meliaceae** The Meliaceae includes 50 genera and 575 species from tropical and sub-tropical regions. *Lansium domesticum* fruits are edible. Timber of high value is obtained from species such as *Swietenia*, mahogany and *Kaha*, African mahogany. *Azadirachta indica*, the neem tree is used as a source of insecticide. *Sandoricum koetjape*, santol and *Lansium domesticum*, Langsat are edible fruit from south east Asia.

**Nitrariaceae** This family has a single genus with 9 species from Africa, Russia and Australia.

**Peganaceae** The Peganaceae has 2 genera with 7 species from temperate and sub-tropical areas of the Mediterranean to Mongolia and the USA and Mexico. This family is the source of a dye.

**Tetradiclidaceae** The Tetradiclidaceae is affiliated with the Zygophyllaceae. *Tetradiclis* is a genus from north Africa and the middle east with 5 species.

**Rutaceae** The Rutaceae, or citrus family has 150 genera and 900 species from temperate to tropical areas worldwide. The fruits of *Citrus* species, orange, lemon and grapefruit are major crops. Some species are important timbers and others are ornamentals. Essential oils are also sourced from this family. Bergamot oil is produced from oranges. *Boronia* species are cultivated for their fragrant oils. *Correa alba* has been used as a tea substitute. Energy from waste biomass may be important from this family.

**Sapindaceae** The Sapindaceae is widespread from temperate to tropical regions with 40 genera and 2000 species. *Litchi chinensis*, lychee; *Nephelium lappaceum*, rambutan; *Nephelium* *mutabile*, pulsan, *Euphoria* *longan*, longan; *Melicoccus* species, Spanish lime and *Blighia sapida*, akee are food species. *Dodanea* are cultivated as ornamentals. *Paullinia cupana* is an ornamental. *Paullina* are also used to produce guarana, and yoco, caffeine containing drinks. *Paullinia* *cururu* is used as an arrow poison.

**Simaroubaceae** The Simaroubaceae has 20 genera and 50 species from tropical and sub-tropical areas. Some species are ornamentals, are used for timber or have medicinal uses.

ASTERIDS

The Astrids are a large group of 10 orders.

Cornales

The Cornales includes 6 families.

**Cornaceae** The Cornaceae or dogwood family has 1-6 genera and about 60 species from tropical mountains and temperate regions. Some species have edible fruits.

**Nyssaceae** The Nyssaceae has 2 genera and 10 species from east Asia and America with some timber species.

**Curtisiaceae** This family has single species, *Curtisia stagnalis*, from South Africa.

**Grubbiaceae** This family includes 2 genera and 5 species from South Africa.

**Hydrangeaceae** The Hydrangeaceae has 10 genera and 115 species from temperate to sub-tropical areas. Hydrangeas are species from this family grown as ornamentals.

**Hydrostachyaceae** This family has only the aquatic genus, *Hydrostachys,* with 22 species from tropical areas.

**Loasaceae** The Loasaceae are tropical plants from 14 genera and 250 species found in Africa and America.

Ericales

The Ericales has 23 families. Examples of 5 families are described here. The other families are Actinidaceae, Balsaminaceae, Clethraceae (reported to contain fructans), Cyrillaceae, Diapensiaceae, Fouquieriaceae, Maesaceae, Marcgraviaceae, Pentaphylaceae, Ternstromiaceae, Sladeniaceae, Polemoniaceae (reported to contain fructans), Primulaceae, Roridulaceae, Sarraceniaceae, Styraceae, Symplocaceae, Tetrameristaceae, Pellicicraceae, Theaceae and Theophrastaceae.

**Ebenaceae** This family has 2 genera with 500 species from mainly tropical environments. The family is source of the hard wood, ebony. *Diospyros kaki*, persimmon is a popular fruit of east Asia. *Diospyros discolor*, velvet apple, also has edible fruit.

**Ericaceae** The Ericaceae has 100 genera with 1350 species from cold climates to tropical (usually at high altitudes in the tropics). Many species such as *Rhododendron* and *Erica* are ornamental. Edible species include; *Vaccinium corymbosum*, blueberry and other *Vaccinium* species, cranberry and huckleberry. *Camellia sinensis* is used to make tea. This family has been reported to store carbohydrates as fructans.

**Lecythidaceae** The Lecythidaceae has 10 genera and 325 species from tropical and sub-tropical South America and Madagascar. *Bertholletia excelsa,* the brazil nut and *Lecythis ollaria*, the sapucaia nut are used as food.

**Myrsinaceae** The Myrsinaceae has 1000 species in 35 genera mainly from the tropics and sub-tropics with some ornamentals.

**Sapotaceae** The Sapotaceae includes 7 genera and 700 species from tropical areas. The family includes many species with edible fruits; *Manilkara zapota*, sapodilla; [*Chrysophyllum cainito*](http://en.wikipedia.org/wiki/Chrysophyllum_cainito)*,* star apple; *Puteria, viridis,* green sapote; *Puteria sapota*, mamaey sapote; *Synsepalum* *dulcificum*, miracle fruit. An isoprene polymer, gutta-percha, was produced from *Palaquium gutta* and used as a plastic for many applications. Some species are used for timber.

EUASTERIDS I

The Eurasterids includes 4 orders and some unplaced families.

**Boraginaceae** The widespread Boraginaceae or borage family has 120 genera and 2000 species. Many species are cultivated. For example, *Borago* *officinalis*, borage is cultivated as a culinary or medicinal herb and as source of oil. This family has been reported to store carbohydrates as fructans.

**Icacinacea** The Icacinaceae includes 52 genera and 400 species from mainly tropical areas. Many species are used for timber.

**Oncothecaceae** The *Oncotheca* is a genus with 2 species from New Caledonia.

**Vahliaceae** This is a genus with 5 species found from south west India to South Africa.

Garryales

**Eucommiaceae** This is a single species, *Eucommia ulmoides*, from China.

**Garryaceae** The sole genus, *Garrya*, has 18 species from USA, central America and the West Indies. Ornamental species have been bred.

**Aucubaceae** The Aucubaceae has one genus with 3 species found from the Himalayas to Japan.

Gentianales

The Gentianales includes 5 families.

**Apocynaceae** The widespread Apocyanaceae has 164 genera and 1500 species.

The family has a range of uses: *Rauvolfia* species are used as sources of pharmaceuticals such as quinine and alkaloids and others are a source of cardiac glycosides. *Carissa congesta* has edible fruit.

**Gelsemiaceae** The family has 2 genera and 11 species from tropical regions.

**Gentianaceae** The Gentianaceae includes 80 genera and 900 species. Many species are ornamentals and some are a source of flavourings.

**Loganiaceae** This family has 1-3 genera and around 30 species.

**Rubiaceae** The Rubiaceae is large and widespread family of 600 genera and 6000 species. Useful species include; *Coffea* species, coffee; *Cinchona* species, quinine and ornamentals such as *Gardenia* and *Randia*.

Lamiales

The Lamiales has 21 families. Details are provided for 4 families. The other families are, Acanthaceae, Bignoniaceae, Byblidaceae, Calceolariaceae, Carlemanniaceae, Gesneriaceae, Lentibulariaceae, Martyniaceae, Orobanchaceae, Paulowniaceae, Pedaliaceae, Phrymaceae, Plocospermataceae, Schlegeliaceae, Scrophulariaceae, Stilbaceae and Tetrachondraceae

**Lamiaceae** The Lamiaceae has 210 genera and 3500 species. The family includes many familiar culinary herbs such as [basil](http://en.wikipedia.org/wiki/Basil_(herb)), [mint](http://en.wikipedia.org/wiki/Mentha), [rosemary](http://en.wikipedia.org/wiki/Rosemary), [sage](http://en.wikipedia.org/wiki/Common_sage), [marjoram](http://en.wikipedia.org/wiki/Marjoram), [oregano](http://en.wikipedia.org/wiki/Oregano) and [thyme](http://en.wikipedia.org/wiki/Thyme). Some species are ornamentals or sources of essential oils.

**Oleaceae** The Oleaceae has 900 species in 25 widespread genera. *Olera* *europaea*, the olive is used as a source of edible oil. Other species in this family are used for timber.

**Plantaginaceae** This family has 3 genera with 270 species.

**Verbenaceae** This family has 90 genera and 3000 species found widely. *Tectona grandis*, teak is an important timber species.

 Solanales

The Solanales has 5 families.

**Convolvulaceae** This widespread family has 55 genera and 1650 species including ornamentals. *Ipomoea batatas*, sweet potato is an important food crop.

**Montiniaceae** This is 2 or 3 genera with 4 species from tropical east Africa and Madagascar.

**Hydroleaceae** This is single genus, *Hydrolea*, with several species.

**Solanaceae** The Solanaceae or potato family includes 95 genera and 2000 species and is widespread and concentrated in South America and Australia. *Solanum tuberosum*, potato; *Solanum melongena*, aubergine; *Lycopersicon esculentum*, tomato; *Capsicum* species, paprika, chillies, red, green and sweet peppers; *Physalis peruviana*, cape gooseberry; *Nicotiana* *tabacum*, tobacco are well known species of economic importance. Some species have medicinal uses. *Cyphomandra betacea*, tamarillo or tree tomato, originally from South America is now grown widely. Some central Australian species are being developed as novel food crops. This family contributes many important food crops and remains a good option for development of new food crops.

**Sphenocleaceae** This is single tropical genus with 2 species.

EUASTERIDS II

The Euasterid II group includes 4 orders and some unplaced families (one example described). Other unplaced families are Columelliaceae, Desfontainiaceae, Eremosynaceae, Escalloniaceae, Paracryphiaceae, Polyosmaceae, Sphenostemonaceae and Tribelaceae.

**Bruniacea** This is a family of 12 genera and 75 species from South Africa.

Apiales

The Apiales has 8-10 families. Details are provided for 3 examples. The other families are Aralidiaceae, Griseliniaceae, Mackinlayaceae, Melanophyllaceae, Myodocarpaceae, Pennantiaceae, and Torricelliaceae

**Apiaceae** The Apiaceae has 300 genera and 3000 species Food species include; *Anethum graveolens*, [dill](http://en.wikipedia.org/wiki/Dill); *Apium graveolens*, [celery](http://en.wikipedia.org/wiki/Celery); *Carum carvi*, caraway; *Coriandrum sativum,* [coriander](http://en.wikipedia.org/wiki/Coriander); *Cuminum cyminum,* cumin; *Daucus carota,* [carrot](http://en.wikipedia.org/wiki/Carrot); *Foeniculum vulgare,* [fennel](http://en.wikipedia.org/wiki/Fennel); *Pastinaca sativa* p[arsnip](http://en.wikipedia.org/wiki/Parsnip) and *Petroselinum crispum*, parsley. The Apiaceae are widely planted as companion plants to protect crops from insects. The 6 carbon sugar apiose is found in this family and can be prepared from a flavanoid glycoside in parsley seed.

**Araliaceae** The Araliaceae includes 49 genera and 700 widespread species including some ornamentals. The roots of *Panax ginseng* are a source of ginseng, used medicinally.

**Pittosporaceae** This family has 10 genera and 200 species including some that are edible (e.g.*Billardiera species*).

Aquifoliales

The Aquifoliales includes 5 families one of which is described.

Aquifoliaceae The widespread holly family includes 2 genera and species with 600 species that are a source of timber.

Asterales

The Asterales contains 11 families. Details are provided for 4 families. The other families are Alseuosmiaceae, Argophyllaceae, Calyceraceae (reported to contain fructans), Lobeliaceae (reported to contain fructans), Menyanthaceae (reported to contain fructans), Pentaphragmataceae, Phellinaceae, Rousseaceae and Donatiaceae

**Asteraceae** The widespread Asteraceae or daisy family is the largest family of flowering plants with 1600 genera and 23,000 species. Food species include; [*Lactuca sativa*](http://en.wikipedia.org/wiki/Lactuca_sativa), lettuce, [*Cichorium*](http://en.wikipedia.org/wiki/Cichorium), chicory, [*Cynara scolymus*](http://en.wikipedia.org/wiki/Cynara_scolymus), globe artichoke, [*Helianthus annuus*](http://en.wikipedia.org/wiki/Helianthus_annuus), sunflower, [*Carthamus tinctorius*](http://en.wikipedia.org/wiki/Carthamus_tinctorius), safflower and [*Helianthus tuberosus*](http://en.wikipedia.org/wiki/Helianthus_tuberosus), Jerusalem artichoke. The non structural storage carbohydrates in this family are polymers of fructose (fructans) rather than starch (a glucose polymer). *Tagetes minuta*, is used to produce marigold oil as flavouring. *Echinacea purpurea* and many other species are used medicinally. Some species are used for herbal teas. Many species are ornamentals.

**Campanulaceae** The Campanulaceae includes 90 genera and 2000 species mainly from northern temperate regions with some significant ornamentals. This family has been reported to store carbohydrates as fructans.

**Goodeniaceae** The Goodeniaceae includes 11 genera and 300 species from Australia, Malaysia, south east Asia, New Zealand, West Indies, South America and Africa with many ornamental species. This family has been reported to store carbohydrates as fructans.

**Stylidiaceae** The Stylidaceae or trigger plant family has 5 genera and 150 species from Australian, south east Asia, Malaysia, New Zealand and South America. This family has been reported to store carbohydrates as fructans.

Dipsacales

The Dipscales contains 7 families.

**Adoxaceae** The Adoxaceae has 3 genera and 3 species from northern temperate areas.

**Caprifoliaceae** The widespread Caprifoliaceae or honeysuckle family has 12 genera and more than 330 species including ornamentals.

**Diervillaceae** The Diervillaceae includes 2 genera and 13 species with ornamental species.

**Dipsacaceae** The Dipsaceae has 14 genera and 150 species from Europe, Asia and Africa with some ornamentals.

**Linnaeaceae** This family has 5 genera and 36 species with ornamentals.

**Morinaceae** The Morinaceae has 3 genera and 13 species from Europe and Asia.

**Valerianaceae** This widespread (except Australia) family includes 8 genera and 400 species with some ornamentals and some medicinal use.

These descriptions of the families of seed plants provide an introduction and should be a starting point in efforts to more systematically find new uses for plants. Families with food species may yield additional related food species with modern approaches to domestication. These have the potential to expand the range of environments in which the foods can be produced. This could be especially valuable in the event of significant climate change. The best sources of energy species are not so clear. The current species under consideration as biofuel crop species are options however any species with a high biomass yield in the target environment should be considered. More trials of a wide range of species are required to provide the data necessary for appropriate energy species selection.

**References**

Chase, M. (2005) Relationships between the flowering plant families. In *Plant diversity and evolution,* (Henry, R.J., ed), CABI, p 7-23

Henry, R.J. (2005) *Plant Diversity & Evolution: Genotypic & Phenotypic Variation in Higher Plants.* Oxon UK: CABI Publishing. pp 332.

Hill, K. (2005) Diversity and evolution of gymnosperms, In *Plant diversity and evolution,* (Henry, R.J. ed), CABI, pp 25-44.
